# Ultrarapid Inductive Rewarming of Vitrified Biomaterials with Thin Metal Forms

**DOI:** 10.1007/s10439-018-2063-1

**Published:** 2018-06-19

**Authors:** Navid Manuchehrabadi, Meng Shi, Priyatanu Roy, Zonghu Han, Jinbin Qiu, Feng Xu, Tian Jian Lu, John Bischof

**Affiliations:** 1 Department of Mechanical Engineering, University of Minnesota, 111 Church Street SE, Minneapolis, MN 55455 USA; 2 Department of Biomedical Engineering, University of Minnesota, 111 Church Street SE, Minneapolis, MN 55455 USA; 3 School of Energy and Power Engineering, Xi’an Jiaotong University, Xi’an, 710049 People’s Republic of China; 4 Bioinspired Engineering and Biomechanics Center (BEBC), Xi’an Jiaotong University, Xi’an, 710049 People’s Republic of China; 5 The Key Laboratory of Biomedical Information Engineering of Ministry of Education, School of Life Science and Technology, Xi’an Jiaotong University, Xi’an, 710049 People’s Republic of China; 6 State Key Laboratory for Strength and Vibration of Mechanical Structures, School of Aerospace, Xi’an Jiaotong University, Xi’an, 710049 People’s Republic of China

**Keywords:** Vitrification, Rewarming, Ultrarapid warming, Nanowarming, Skin depth, RF heating, Tissue preservation

## Abstract

Arteries with 1-mm thick walls can be successfully vitrified by loading cryoprotective agents (CPAs) such as VS55 (8.4 M) or less concentrated DP6 (6 M) and cooling at or beyond their critical cooling rates of 2.5 and 40 °C/min, respectively. Successful warming from this vitrified state, however, can be challenging. For example, convective warming by simple warm-bath immersion achieves 70 °C/min, which is faster than VS55’s critical warming rate of 55 °C/min, but remains far below that of DP6 (185 °C/min). Here we present a new method that can dramatically increase the warming rates within either a solution or tissue by inductively warming commercially available metal components placed within solutions or in proximity to tissues with non-invasive radiofrequency fields (360 kHz, 20 kA/m). Directly measured warming rates within solutions exceeded 1000 °C/min with specific absorption rates (W/g) of 100, 450 and 1000 for copper foam, aluminum foil, and nitinol mesh, respectively. As proof of principle, a carotid artery diffusively loaded with VS55 and DP6 CPA was successfully warmed with high viability using aluminum foil, while standard convection failed for the DP6 loaded tissue. Modeling suggests this approach can improve warming in tissues up to 4-mm thick where diffusive loading of CPA may be incomplete. Finally, this technology is not dependent on the size of the system and should therefore scale up where convection cannot.

**Electronic supplementary material:**

The online version of this article (10.1007/s10439-018-2063-1) contains supplementary material, which is available to authorized users.

## Introduction

The use of low temperatures to indefinitely bank and store tissues for eventual transplantation is a main goal of the field of cryobiology.^15^,^19^,^24^ This approach, often termed cryopreservation, has been successfully used for long-term storage of cells, aggregates, and some smaller tissues.^10^,^15^,^19^,^35^ However, the availability of many tissues and almost all organs is limited due to several important bottlenecks, including the need for faster warming in larger and thicker tissue systems.^11^,^12^,^16^,^18^

One of the best options for cryopreservation of tissues and larger systems involves vitrification or storage in a glassy state that avoids ice crystal damage. This requires tissues and other biomaterials (i.e., suspensions) to be loaded with cryoprotective agents (CPAs) to block ice crystal formation during cooling to temperatures below the glass transition temperature of the CPA, usually − 140 °C.^10^

CPA toxicity can generally be avoided by using lower concentration CPAs, but this means that the critical warming rate (CWR) increases from 55 °C/min for 8.4 M VS55 to 185 °C/min for 6 M DP6^7^,^22^,^34^ to avoid devitrification during warming.^7^,^8^,^18^,^22^,^34^ Similarly, as tissue thickness increases, longer loading time (hours) would be needed to equilibrate the distrubution of CPA in the tissue. As this time increases beyond normal loading times (4–5 steps of roughly 15 min each), the potential for toxicity and therefore lower viability at the edge of the tissue being loaded will increase. Therefore, all tissues (thick or thin) are loaded with the least amount of CPA over the least time to avoid toxicity. As a result, there will always be a lower concentration in the center of the loaded tissue which, especially in the case of thicker tissue, will necessitate faster rates of warming to successfully avoid crystallization damage.

To address these issues, microwave warming^9^,^17^,^23^,^25^,^26^,^36^ and nanowarming with nanoparticles^8^,^18^ have been proposed to achieve faster warming of tissues than convection. Microwave warming, however, can be non-uniform, resulting in “hot spots” which can drive subsequent cracking and/or thermal runaway. Moreover, its application varies by size and shape within a system.^3^,^4^,^9^,^25^ Alternatively, nanowarming adds biocompatible magnetic (iron oxide) nanoparticles to the CPA prior to vitrification and storage. Rewarming is achieved within an electromagnetic coil producing a uniform radiofrequency field that inductively heats the nanoparticles and then, by extension, the whole system.^8^,^18^ This technology has achieved uniform warming rates up to 100 °C/min for porcine tissues regardless of volume,^18^ and has successfully warmed ~ 1 mm arteries loaded with VS55 in 1–50 mL systems. Nevertheless, the rates necessary to successfully warm DP6 solutions, loaded arteries, or thicker arteries (i.e., aorta) are difficult to achieve with achievable rates from convection or nanowarming.

**Figure 1:**
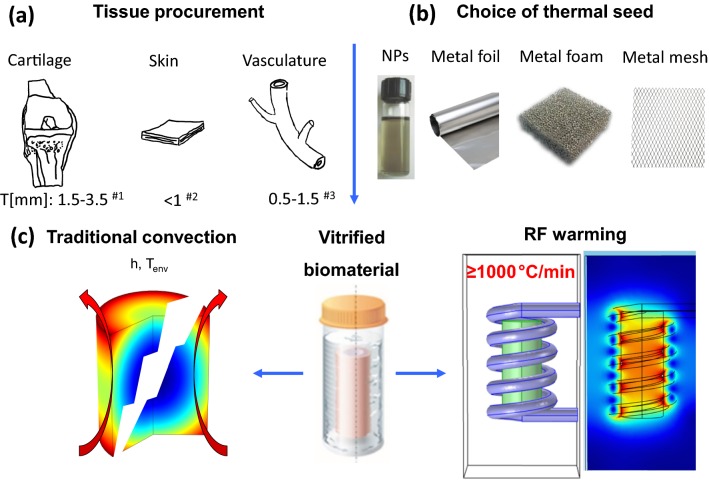
Schematic of planar and annular tissues that can benefit from the ultrarapid warming method. Ultrarapid warming can be deployed in solutions or tissues with several mm thickness, especially with planar or cylindrical geometries (a). Both ultrarapid warming (with foam/foil/mesh) and nanowarming (with nanoparticles) relies on coupling the alternative magnetic field to the metallic substance, but an order of magnitude higher rate is possible with ultrarapid warming approach (b). Both approaches ensure homogenous and fast warming rates to avoid devitrification and cracking can occur with convection (c). The references in (a) are Refs. 18, 27, and 28.

In this study, we developed an ultra-rapid volumetric heating method to heat solutions or tissue systems at ≥ 1000 °C/min, which is a rate that is an order of magnitude faster than convection, nanowarming or the CWR of DP6 and VS55.^8^,^18^,^22^ Solutions or tissues were vitrified and placed in contact with thin metal forms (foam, foil, or mesh) as shown in Fig. 1. The alternating magnetic field induced eddy currents in the metal with concurrent resistive losses that heated the sample from within (Figure S2A). As a demonstration of use, this approach was then used to successfully rewarm DP6 loaded carotid arteries where convection failed.

## Materials and Methods

### Preparation of Thin Metal Forms: Foam, Foil, and Mesh

In this study, metal forms consisted of copper foam (YiYang Foammetal New Materials Co., Ltd., Hunan, China), 100-*µ*m thick aluminum foil (Reynolds Wrap, Lake Forest, IL), and 500-*µ*m thick nitinol mesh (Boston Scientific, Saint Paul, MN) structures as shown in Tables 1 and 2. These choices were made to test a range of metals with variable electrical properties and ease of shaping.

**Table 1:** Thermal and electrical properties of metals used in study. Theoretical SAR is calculated for a solid rod with the diameter of 0.965 mm heated in an RF system working at frequency of *f* = 360 kHz and magnetic field intensity of *H*_0_ = 20 kA/m.

Metal	Skin depth (*δ*) (*μ*m)	Resistivity (*ρ*_e_) (Ω.m)	Electrical conductivity (*σ*) (S/m)	Relative permeability (*μ*_r_) (–)	Density (*ρ*) (g/cc)	Volumetric SAR_The_ (W/cm^3^)	SAR_The_ (W/g)
Copper	140	1.68 × 10^−8^	5.96 × 10^7^	0.9999	8.96	936	105
Aluminum	108	2.82 × 10^−8^	3.50 × 10^7^	0.9994	2.70	1221	450
Nitinol	760	82 × 10^−8^	0.12 × 10^7^	1.0020	6.50	6550	1000

**Table 2:** Physical properties of copper foam, aluminum foil and nitinol mesh.

Thickness (*μ*m)	Mass (g)	Width (cm)	Length (cm)	Volume (cm^3^)	Density (g/cm^3^)
200	2.50	0.965	2.29	1.67	0.98
100	0.79	0.940	2.34	1.62	0.48
500	0.77	0.050	2.25	0.12	0.50

HR (°C/min)	SAR_Exp_ (W/cm^3^)	Metal type and structure		SAR_Exp_ (W/g)	SAR_Exp_/SAR_The_ (%)
1200	80	Copper foam		82	80
1000	200	Aluminum foil		415	92
330	480	Nitinol mesh		960	96

Heating rates and experimental SARs are calculated from the linear portion of the heating profile

**Figure 2:**
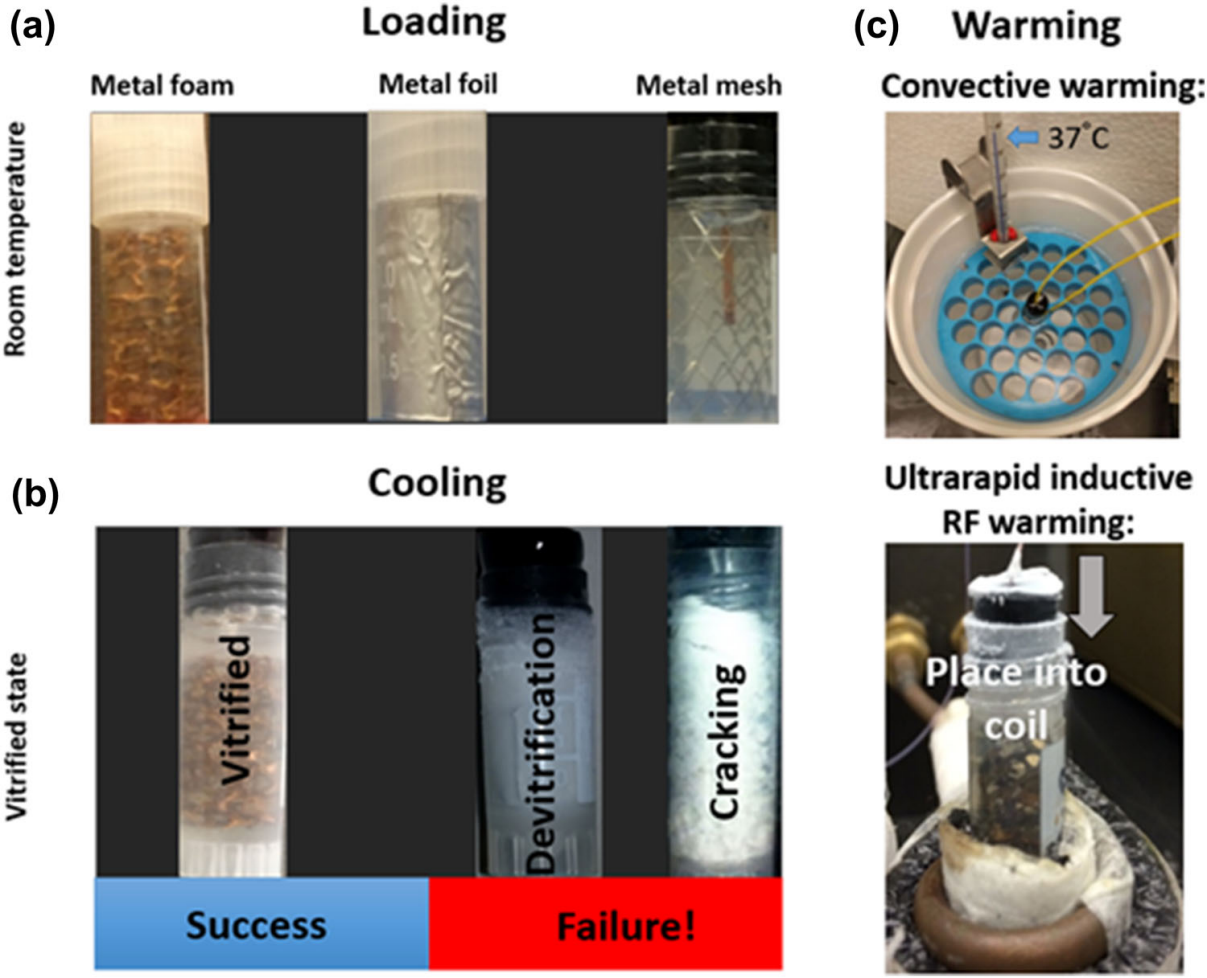
Ultrarapid warming steps using metal forms (foams, foils or meshes). (a) Loading of VS55 or DP6 in a 1.8-mL cryovial containing copper foams, aluminum foil or nitinol mesh at room temperature. (b) Success and failure of cooling solutions to sub-glass transition temperature − 140 °C. (c) Rewarming vitrified solutions by convection or inductive RF heating (ultrarapid warming).

Preliminary tests were performed with copper foams with 20 pores per inch and 89percent porosity. These were formed by wire electrical discharge machining into a cylindrical shape with diameter and height of 9.8 and 23 mm, respectively. Two fluoroptic probes (Qualitrol Company LLC, Fairport, NY) were used to monitor the temperature at the center and edge of the foam with a vertical position of 30 mm from the top of a 1.8-mL cryovial. The metal foams were placed in the cryovial (Cole Parmer, Vernon Hills, IL), and CPA was added until reaching 1.8-mL volume (Fig. 2a). The metal foil and mesh were formed into a cylindrical shell (annulus) shape with properties mentioned in Table 2. Cooling and heating under these conditions without the presence of an artery or tissue are understood to represent limiting cases.

### Cooling Approaches

The two CPAs, VS55 and DP6, have very different critical cooling rates, so two separate cooling protocols were used to achieve vitrification at rates that exceed the critical cooling rate (Fig. 2b).

#### Fast and Direct Cooling (DP6)

DP6 solution requires a high critical cooling rate (− 40 °C/min) for vitrification. To achieve this, the 1.8-mL cryovials loaded with the metal and CPA were lowered into a large flask filled with liquid nitrogen and held in the vapor phase (− 160 °C) just above the surface of the liquid. Temperature was monitored using two fluoroptic probes placed at the center and edge of the vial (Qualitrol Company LLC, Fairport, NY) connected to a T/GUARD 405 temperature monitoring system (Neoptix, Canada). One of the probes measured the centerline temperature of the CPA solution in the vial while the other was placed on the outside near the vial wall. When the center reached − 115 °C, the vial was allowed to anneal by taking the cryovial out of the flask for 5–7 s to allow the center and the edge of the CPA inside the cryovial to equilibrate and stabilize. By performing this just above the glass transition temperature (− 123 °C for VS55^20^ and − 119 °C for DP6^22^), residual thermal stresses are reduced, thereby lowering the chance of cracking when the sample transitions into a glass. Finally, the samples were cooled to − 140 °C, and monitored for any cracking (more than 90% of samples achieved vitrification without cracking). A number of these successfully vitrified samples were then either placed in the RF system or convective water bath to compare warming processes.

#### Slow and Controlled Cooling (VS55)

VS55 has a lower critical cooling rate (− 2.5 °C/min) than DP6, so a multi-flask cooling method for a 1.8-mL system was used as previously described.^8^,^18^ In brief, the cryovials were placed in a series of concentric, successively larger containers, with liquid nitrogen filling the outside of most containers. This layering of containers provided thermal barriers for heat transfer between the liquid nitrogen and the cryovial and slowed down the heat transfer rate. To monitor the temperature during cooling, two fluoroptic probes were positioned in the center and on the edge of the cryovial as described in the DP6 cooling section above. Once the center reached − 115 °C, the sample was annealed for 5–7 s and finally cooled to − 140 °C. The vast majority of these samples were vitrified and non-cracked. A number of these were then placed either in an RF system or convective water bath to compare warming processes.

### Warming Approaches

#### Convective Warming

The vitrified samples were transferred from the liquid nitrogen container and immersed into a 37 °C water bath while the temperature variation was recorded using fluoroptic probes placed in the middle and edge of the sample (Fig. 2c—top).

#### Ultrarapid Warming

As shown in Fig. 2c—bottom, 1.8-mL vitrified samples consisting of metal (foam, foil and mesh) loaded in DP6 and VS55 at − 140 °C were quickly transferred into the coil of a 1-kW Hotshot inductive heating system. The RF system has a 2.5–turn water-cooled copper coil (Ameritherm Inc., Scottsville, NY), and experiments were carried out at a magnetic field strength of 20 kA/m (peak, volume-averaged field strength) and frequency of 360 kHz. The cryovial was placed within a Styrofoam container within the coil to lessen direct loss to the environment. The time of RF exposure was characterized for each case of metal forms (copper foam, aluminum foil and nitinol mesh) to ensure the final temperature of − 20 °C (near melt of CPA) was reached prior to turning off the field. Typically, the temperature would then continue to rise more slowly to room temperature prior to any further studies. To assess the warming efficacy, we considered warming rates from − 140 °C vitrified state to − 20 °C, where CPA is liquid and the low temperature leads to a reduction in toxicity. We noted that the resulting heat generation or specific absorption rate (SAR) depends on the metal type, shape, structure, weight, volume and orientation of placement in RF coil. Therefore, samples from different metal forms warm at different rates and lead to different volumetric SAR (W/cm^3^) or mass-based SAR (W/g) as shown in Table 2. The sample temperature was achieved continuously at a frequency of 5 Hz by means of fluoroptic probes.

### Viability Studies

For viability experiments, we chose to work with aluminum foil warming due to ease of deployment in DP6 and VS55 samples and compared this to convective warming controls. Porcine arteries were obtained postmortem from skeletally immature domestic Yorkshire cross farm pigs (65–80 kg, aged 16–18 weeks). Arteries were removed within 30 min of death following Institutional Animal Care and Use Committee (IACUC) approved protocols at University of Minnesota. The animals were sacrificed as part of other IACUC approved studies at the Visible Heart Lab and the arteries were considered bona fide excess. Arteries were submerged in a Krebs–Henseleit buffer and placed on ice before being transported to our laboratory. Upon receipt (hours later), arteries were dissected to reproducible segments ~ 1-cm height. Fresh artery segments were rinsed with growth media [Dulbecco’s modified Eagle’s medium (Thermo Fisher) with 1% antibiotic-antimycotic (Thermo Fisher)], and cleared of fatty tissue. Carotid arteries were sectioned into 1 cm-long segments with inner diameters of 4–6 mm and wall thicknesses 1–2 mm. Experiments were carried out on several independent days with 3–4 arteries per test and 4–6 slices per artery for ultrarapid warming and convective warming tests.

Viability was assessed by incubation with 10% alamarBlue (Thermo Fisher) media solution at 37 °C for 3 h before (control) and after any warming experiments. Fluorescence was read on a plate reader (Synergy HT, BioTek) at 590 nm from an aliquot of the media to establish a baseline. For arteries undergoing ultrarapid or convective methods, tissues were stepwise loaded with CPA as previously published.^1^,^18^ Once the arteries experienced the final step of loading at full-strength VS55 or DP6, the aluminum foil was placed against the interior (i.e., luminal) and exterior artery walls to create a sandwich, and the remainder was filled with the CPA. All samples were successfully vitrified by the protocols explained above and equilibrated at − 140 °C prior to transfer to the warming apparatus.

Ultrarapid rewarming was achieved by either inductive RF warming at 20 kA/m, 360 kHz, or by convective warming using 37 °C water bath immersion, considered here as a gold- standard control. After warming to room temperature (18–20 °C), the VS55 and DP6 was step-wise removed as previously reported.^1^ After the removal of the CPA, the tissue segments were sectioned into small pieces and incubated with fresh media at 37 °C for one hour (recovery) and then incubated with 10% alamarBlue for 3 h and compared with fresh controls. The viability of each tissue piece was normalized to fresh control. Raw results are presented as the mean ± standard error of relative fluorescence units (RFU) after correction to RFU/mg dry weight prior to normalization.

#### Heat Transfer Modeling

**Figure 3:**
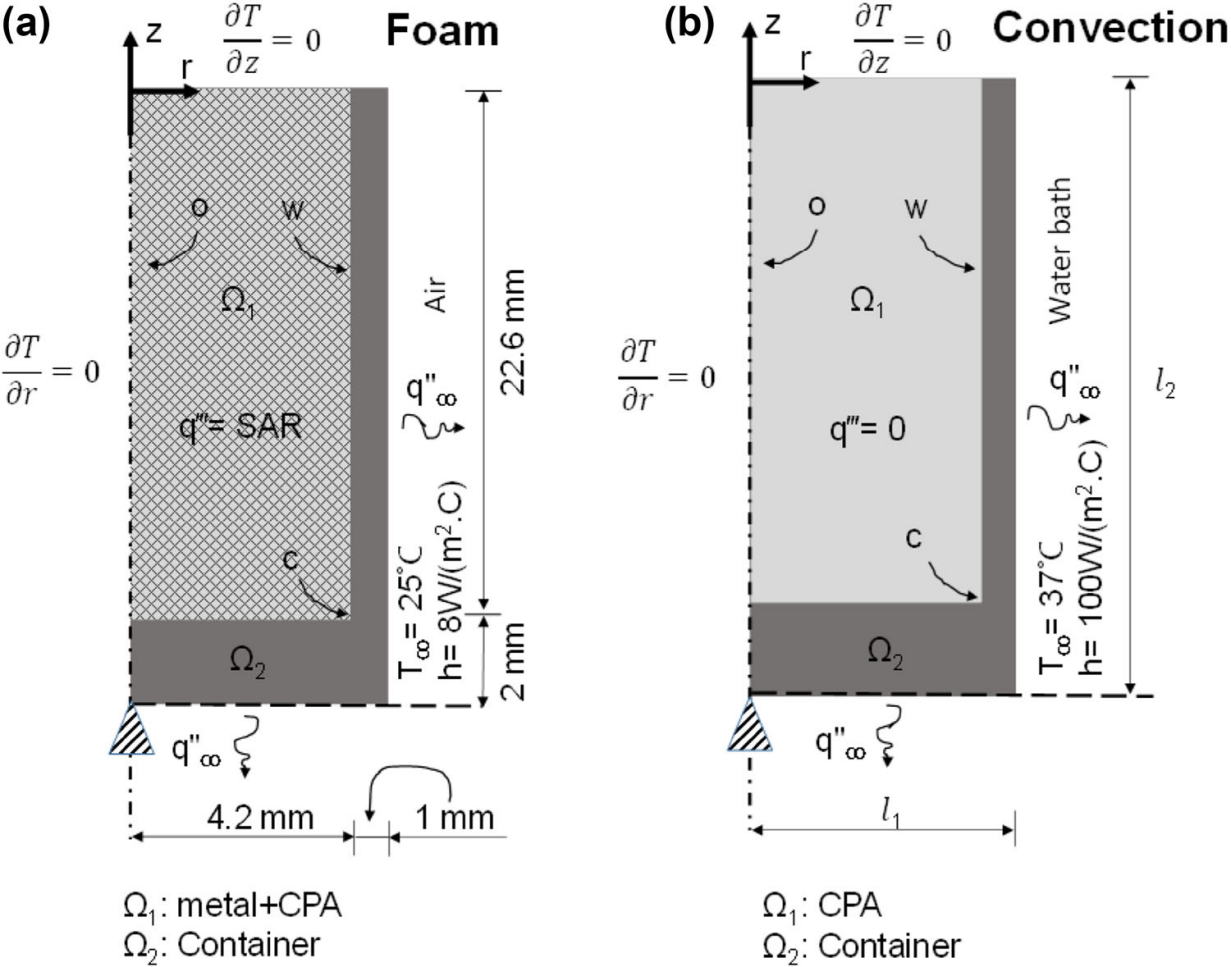
Schematic of the combined thermal and solid mechanics modeling. Boundary conditions and initial conditions are given for both ultrarapid warming of solutions using foam (a) and convective warming in water bath (b).

Temperature modeling was approached using a 2-D cylindrical energy equation for solving the heat-transfer problem. Different solid domains were assigned as shown in Fig. 3 for metal, cryovial and tissue (when present), and the heat diffusion equation was solved:

1 $$\frac{1}{r}\frac{\partial }{\partial r}\left( {kr\frac{\partial T}{\partial r}} \right) + \frac{\partial }{\partial z}\left( {k\frac{\partial T}{\partial z}} \right) + {\text{SAR}} = \rho c_{\text{p}} \frac{\partial T}{\partial t} ,$$

where *k* represents the thermal conductivity, *ρ* the density, *c*_p_ the specific heat capacity, *T* the temperature, and SAR (W/m^3^) the volumetric heat generation rate from the metals due to RF heating. Initial and boundary conditions and other parameters are listed in the Supplemental Material under “heat-transfer modeling” and are listed for specific cases in Fig. 3.

The solution domain consists of two concentric cylinders. In case of metal foam (Fig. 3a), the inner cylinder Ω_1_ is made up of CPA and foam. Thermal properties for the different domains are listed in Table S2A. In the case of CPA and foam in the middle of the cylinder, the properties are estimated by mass averaging using this equation:

2 $$X_{\text{effective}} = \phi X_{\text{CPA}} + (1 - \phi )X_{\text{metal}} ,$$

where *X*_CPA_ and *X*_metal_ are the corresponding properties for the pure CPA and pure metal, respectively, and *ϕ* is the mass porosity of the metal foam. Volumetric heat generation is confined in the domain Ω_1_, and domain Ω_2_ mimics the polypropylene cryovial.

**Figure 4:**
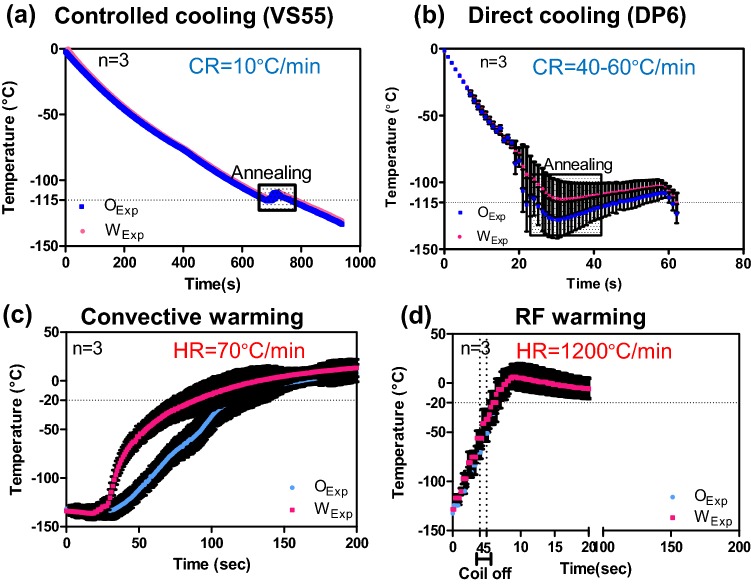
Experimental measurement of cooling and warming rates of vitrified vials. The cooling response of CPA and metal implanted cryovials are shown for VS55 (a) and DP6 (b). The respective rates of 10 and 40–60 °C/min exceed the critical cooling rates needed for VS55 (2.5 °C/min) and DP6 (40 °C/min). The warming responses during convection (c) and ultrarapid warming (d) are shown to achieve rates of 70 and 1200 °C/min, respectively. In (d), the RF coil was shut down at − 50 °C to avoid overheating the sample.

Figure 3b describes the case of convective warming. In this model, the domain Ω_1_ consists of only CPA with no internal heat source, and warming is achieved only by boundary heating. The boundary conditions and initial condition for the heat-transfer problem are also indicated in Fig. 3. In both Figs. 3a and 3b, the top and left (symmetry) boundaries are assigned adiabatic conditions, while the bottom and right side of the container are assigned convective conditions. The free convection heat transfer coefficient in air (Fig. 3a) was taken as 8 (W/(m^2^·K)) based on an empirical correlation from Incropera and DeWitt,^14^ whereas in water (Fig. 3b) it is assumed to be 100 (W/(m^2^·K)) based on the ability to fit the experimentally determined convective heating response shown in Fig. 4c.

#### Solid Mechanics Modeling

While the heat transfer is unaffected by solid mechanics, the inverse is not true. Specifically, the mechanical response of the material is driven by thermal strain caused by temperature distribution within the domains as discussed in detail in the Supplemental Material. To assess this coupling, mechanical and thermal properties were used in simulations as listed in the supplementary Table S2A.^5^,^6^,^33^ The CPA in domain Ω_1_ was modeled as a viscoelastic linear Maxwell fluid with a single-branch spring-dashpot behavior.^32^ The viscosity of the fluid increases as per supplementary Table S2B with drop in temperature until the fluid behaves as a solid at close to its glass-transition temperature. The total strain rate is calculated as the sum of elastic, creep, and thermal strain rates.^5^,^29^

3 $$\dot{\varepsilon } = \dot{\varepsilon }_{\text{creep}} + \dot{\varepsilon }_{\text{elastic}} + \dot{\varepsilon }_{\text{thermal}}$$

Domain Ω_2_ was the container, and it was set to act as an elastic solid over the temperature range considered. As shown in Figs. 3a and 3b, two different geometries were used corresponding to metal foam + CPA and CPA-only cases. The bottom center of the cylinder was used as a pinned boundary condition, while all other boundaries could move freely. Here, the shear stress is assumed to be negligible, and since the circumferential stress is much smaller than axial stress,^5^ it is not considered in the modeling. The commercial FEA package COMSOL Multiphysics was used for all the numerical heat and mechanics based simulations. In all cases, numerical stability and convergence were ensured as further mesh reduction and discretization left the solution unchanged.

#### Diffusional (CPA) Loading Model

To model CPA loading, a 1D cylindrical annulus model of mass (i.e., CPA) diffusion based on Fick’s 2nd Law, was applied with the concept that the CPA concentration, *C*, is governed by an effective diffusivity, *D* (m^2^/s) in the tissue:

4 $$\frac{1}{D} \cdot \frac{\partial C}{\partial t} = \frac{1}{r} \cdot \frac{\partial }{\partial r}\left( {r \cdot \frac{\partial C}{\partial r}} \right)$$

with the boundary conditions and initial conditions as noted in Supplemental Methods. The method of separation of variables (analytical closed form) was used to obtain an exact solution for *C*(*r, t*) as shown in the Supplemental Material. This closed-form solution was then plotted and visualized using MATLAB (MathWorks).

The value of diffusivity was estimated by fitting the theoretical curve to historical experimental data for VS55 loading into a carotid artery.^18^ More specifically, the boundary conditions were normalized with respect to the external CPA solution concentration at the first 18-min time step. The coefficient of determination, *R*^2^, was used to assess how well the model was able to predict the experimental data. The value of *R*^2^ was estimated as:

5 $$R^{2} \equiv 1 - \frac{{\sum\nolimits_{i} {(y_{i} - f_{i} )^{2} } }}{{\sum\nolimits_{i} {(y_{i} - \bar{y})^{2} } }},$$

where *y*_i_ is the experimental data, *f*_i_ is the theoretical value for the same radius, $\bar{y}$ is the average of experimental data. The range of *R*^2^ is from 0 to 1, with values closer to 1 indicating a better fit of the model to the data.

## Results

Figures 4a and 4b and the images in Fig. 2 demonstrate that we can exceed the critical cooling rates of VS55 (2.5 °C/min) and DP6 (40 °C/min) to achieve vitrified solutions as shown by the clarity of the solution and absence of cracks. Cooling rates measured were 10 °C/min for VS55 and 40–60 °C/min for DP6. Further, the slower VS55-controlled cooling method and the metal foam samples show minimal thermal gradients in the sample (Fig. 4a). Both samples were annealed at roughly − 115 °C, which is just above the glass-transition temperatures of − 123 °C for VS55^20^ and − 119 °C for DP6.^34^

**Figure 5:**
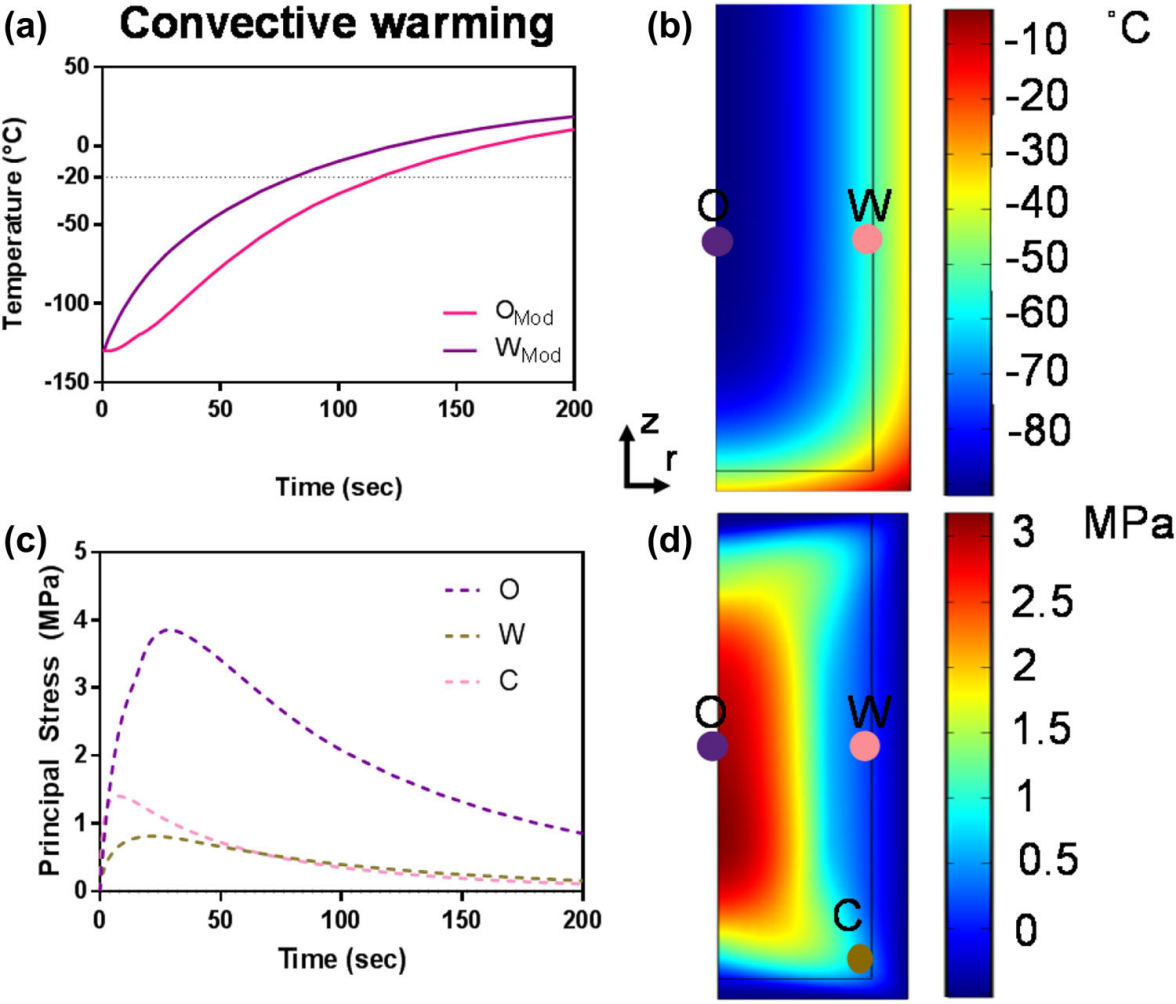
Computational thermal and mechanical stress analysis of convective warming. The simulated thermal (A and B) and stress responses (C and D) to 37 °C water bath convective warming are shown. The maximum thermal gradient of 35 °C during convection results in thermal stress of ~ 4 MPa, which exceeds the yield stress of 3.2 MPa^34^ at the central point O along the axis of symmetry (C and D).

Figures 4c and 4d show an experimental comparison of inductive and convective warming of these vitrified materials from − 140 °C, respectively. Warm-bath immersion leads to convective rates up to 70 °C/min while inductive heating achieves ≥ 1200 °C/min reaching − 20 °C in seconds. Indeed, it was necessary to shut the RF coil off at − 50 °C to avoid overheating of the sample (Fig. 5d). Temperature profiles at the center or origin (O) to wall (W) are similar for ultrarapid warming; however, a significant gradient is shown in the convective water bath (Fig. 4c). These results show our ability to exceed CWR of DP6 and VS55 by ultrarapid warming of copper foams. Similar measurements showing our ability to exceed CCR and CWR were made for other metals in VS55 as shown in the Supplemental Material and summarized in Table 2 and Figure S2.

**Figure 6:**
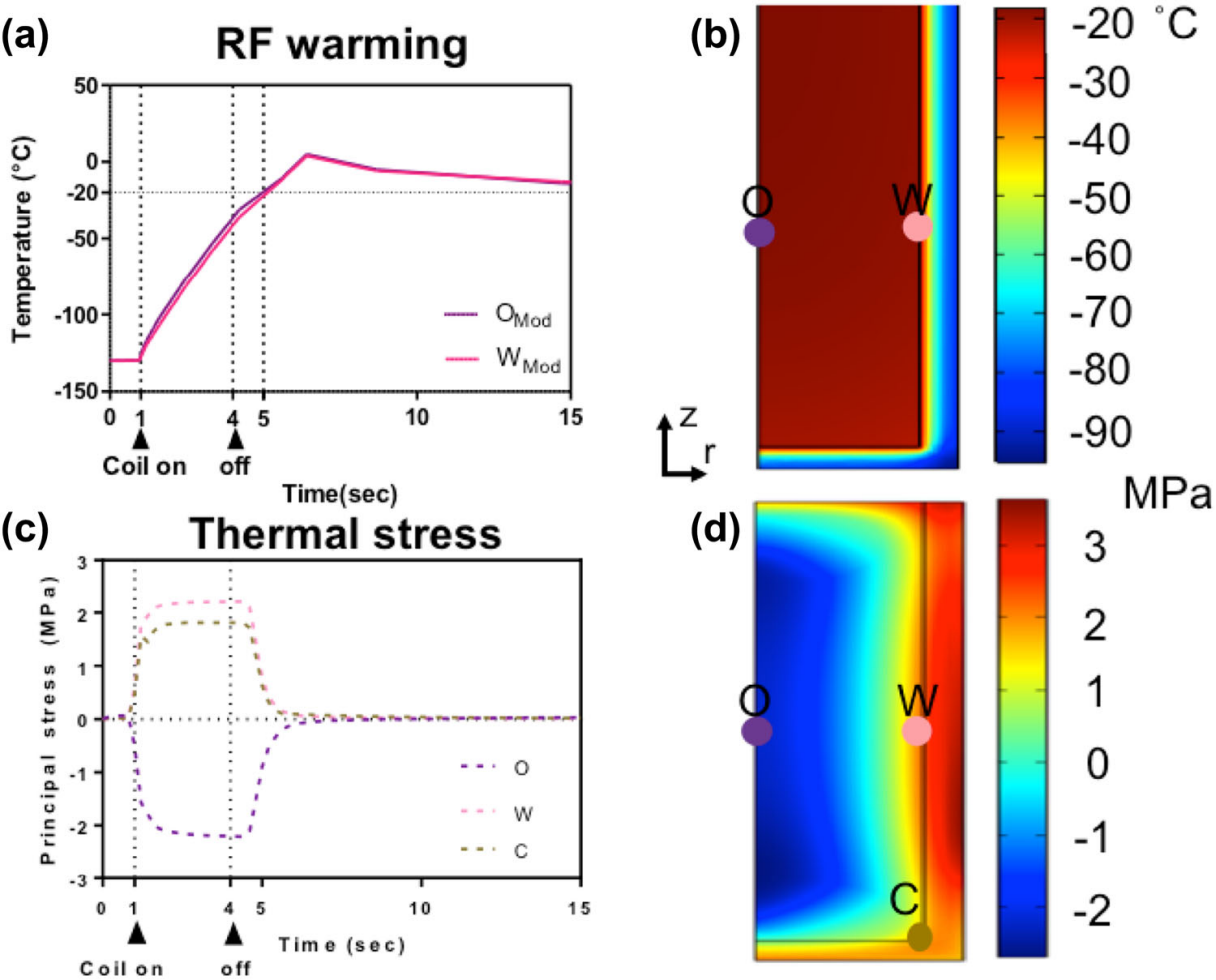
Computational thermal and mechanical stress analysis during ultrarapid warming. The simulated thermal (a and b) and stress responses (c and d) to ultrarapid heating are shown. The positive values in (c) represent tension, while negative values imply compression in the system. Note that the vitrified system would fail at much lower tension than compression; therefore, failure may occur in areas where the positive stress is maximum (e.g., a wall) but still below the yield stress of 3.2 MPa^34^ (d).

The predicted thermal and mechanical response of the systems during convective and inductive ultrarapid warming are shown in Figs. 5 and 6 respectively. For instance, there is a maximum thermal gradient of 35 °C in a convective water-bath method (Figs. 5a and 5b), resulting in thermal stress beyond the yield stress, specifically at the center or origin of ~ 4 MPa, which will likely lead to failure by cracking (Figs. 5c and 5d). On the other hand, the computed thermal response (Figs. 6a and 6b) compares favorably to the experimental warming profiles (Fig. 4d) of a metal foam heating with RF. Figures 6c and 6d illustrate the corresponding thermomechanical stress response. The positive sign in Fig. 6c represents tension, and the negative sign represents compression in a biomaterial. Note that a vitrified biomaterial would fail at much lower tension than compression, so we look for areas where the positive stress is maximum (e.g., at the wall (W) in Fig. 6d). The simulated stress level is 40% less than the critical yield stress of 3.2 MPa, suggesting that failure by cracking is unlikely.

**Figure 7:**
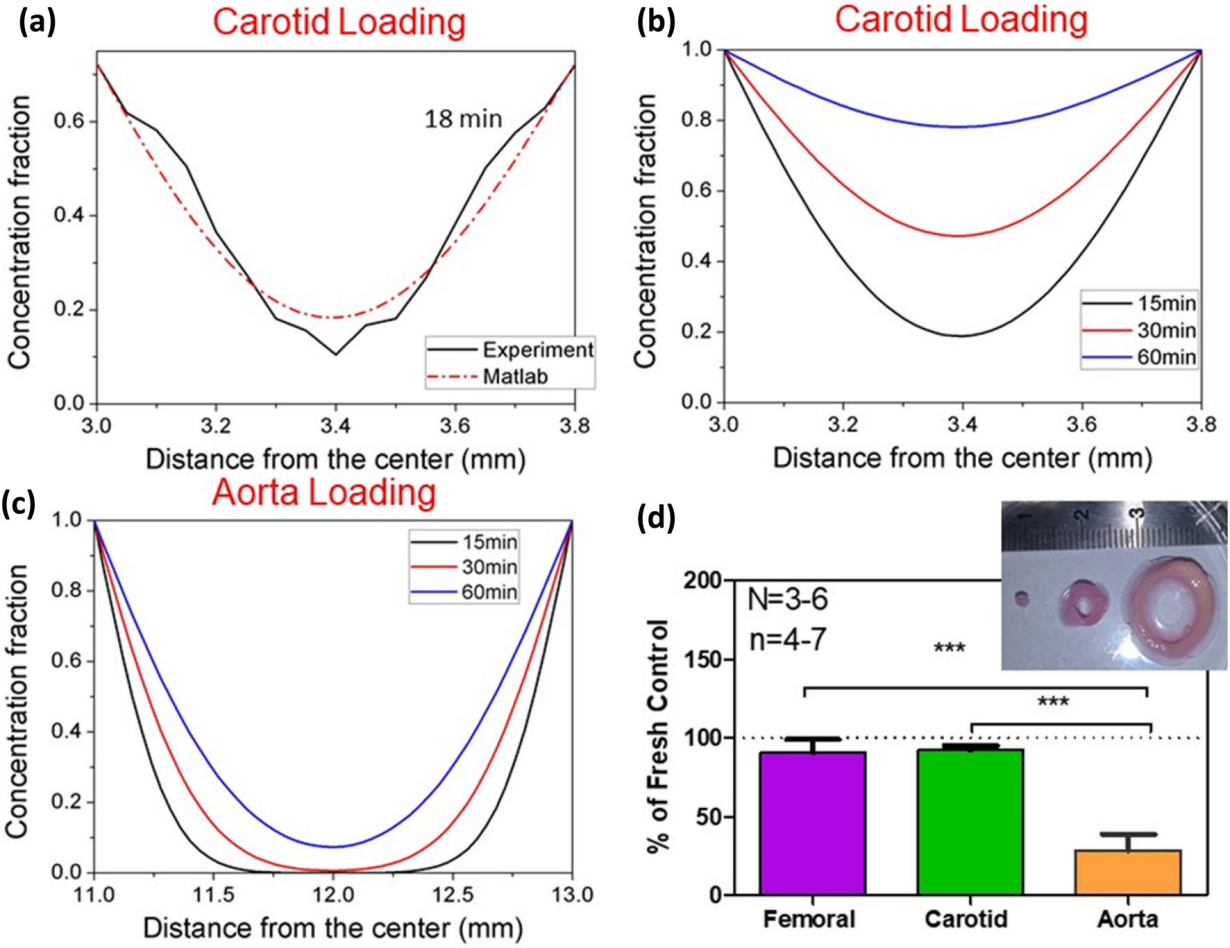
Diffusion loading of VS55 with variable rewarming success in arteries. (a) Historical microCT data of VS55 loading into a carotid artery (0.8-mm thick) at 18 min,^18^ used to extract VS55 diffusivity. (b) Predicted temporal and spatial concentration profiles of VS55 with extracted diffusivity and normalized boundary conditions for the same artery at 15, 30 and 60 min. (c) Predicted temporal and spatial concentration profiles of VS55 in thicker 2 mm aorta at 15, 30 and 60 min. (d) Historical data^18^ post nanowarming in carotid artery and aorta with convection and nanowarming. The viability is reduced for aorta compared to femoral (29 ± 8.2% vs. 90.5 ± 7.4%, *** *p* < 0.001) or to carotid (29 ± 8.2% vs. 91.5 ± 2.4%, *** *p* < 0.001), where *n* = 3–6 is the number of independent experiments, and *n* = 4–7 is the number of segments tested.

Even with an order-of-magnitude higher warming rate, this technique will still be limited by the amount of CPA that can be effectively loaded into the artery. To address this, VS55 diffusive loading into carotid arteries was studied (Figs. 7a–7c), and warming of CPA loaded arteries of variable thickness was attempted experimentally (Fig. 7d). By matching the diffusion equation to the experimental data at 18 min, a mass diffusivity of 3.18 ×10^−11^ m^2^/s was extracted as shown in (Fig. 7a). The final *R*^2^ for the data set at 4 °C at 18 min was 0.94 indicating a good fit.^18^ To assess the impact of differential loading experimentally Fig. 7b shows loading of CPA into a thin carotid artery. Clearly, the viability drops as the amount of CPA that can be delivered to the tissue center is reduced in the aorta. Using the diffusion loading model, spatial and temporal distribution of VS55 concentration within the carotid artery is shown to reach more than 60% in the center after 60 min in Fig. 7b whereas the 2-mm thick aorta reached only 10% loading in the center over the same time frame with the same boundary loading (Fig. 7c). To assess the impact of differential loading, the viability of thin (femoral), average (carotid), and thick (aorta) arteries loaded by the same step protocol was assessed after nanowarming (Fig. 7d). As can be seen, the carotid and femoral arteries (roughly 1-mm thick) had high viability, while the aorta showed low viability. Thus, CPA loading is critical as lower loading leads to lower yields from nanowarming.

One approach to successfully rewarm tissues with less CPA is to increase the warming rate beyond both convection and nanowarming with metal forms. To explore this more generally, the heat generation of copper foams, aluminum foil and nitinol were all predicted and tested. Table 1 summarizes the theoretical SAR predictions based on Stauffer^31^ for a solid rod with a diameter of .965 mm heated in an RF system working at frequency of 360 kHz magnetic field intensity of 20 kA/m. The results show nitinol volumetric heat generation at almost six times that of aluminum and copper. Table 2 summarizes the experimental SAR generation for copper foam, aluminum foil and nitinol mesh.

**Figure 8:**
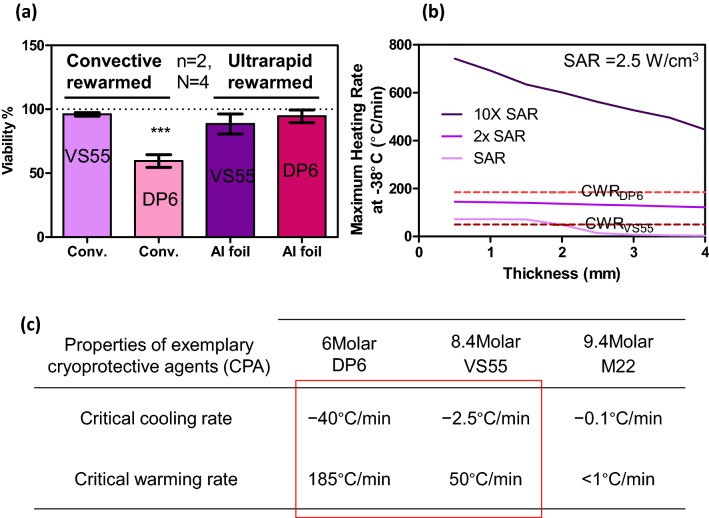
Viability of carotid artery segments after convection and ultrarapid warming. (a) Viability was statistically unchanged after ultrarapid warming of VS55 (88.5 ± 5.5%) or DP6 (94.5 ± 3.5%) loaded arteries vs. convective VS55 controls while convectively warmed DP6 loaded arteries (59.5 ± 3.5%, *p* < 0.001***) proved to be ~ 35% less effective than VS55 convective controls (*n* = 2 independent experiments and *n* = 4 segments per test). (b) Annular model of artery warming with variable SAR and tissue thickness adopted from Manuchehrabadi *et al.*^18^ Here, a typical volumetric SAR generated from magnetic nanoparticles heating (nanowarming) is given as SAR = 2.5 W/cm^3^. This simulation suggests that 10× SAR, achievable by the ultrarapid method, can warm up to 4-mm-thick arteries if deployed in the lumen and around the artery. Some properties of conventionally used CPA are given in (c).

Finally, the viability of warmed carotid artery segments was assessed with varying amounts of CPA loading (Fig. 8a). Importantly, to avoid temperature variations within the artery during ultrarapid warming, the foil was deployed as a sandwich around the artery prior to cooling and warming. After convective warming for DP6-loaded arteries showed a ~ 35% drop in viability vs. VS55-loaded arteries (*p* < 0.05). However, there was no statistically significant viability changes between VS55 or DP6 control arteries after ultrarapid warming. While this demonstrates the ability for ultrarapid warming to recover tissues with sub-optimal CPA penetration (i.e., DP6 carotid), it also suggests that ultrarapid warming may work for thicker tissues. To evaluate this theoretically, we varied the heat generation of the warming method in an annular model of an artery as shown in Fig. 8b.^18^ Here a baseline volumetric SAR of 2.5 W/cm^3^ represents nanowarming. By increasing this SAR by an order of magnitude (i.e., 10× SAR), one obtains a trend that “arteries” or annular tissues up to 4-mm-thick can be warmed at rates beyond the CWRs of VS55 and DP6. Importantly, these SARs and higher are achievable by deploying the metal forms in the lumen and around the outside of the artery (Table 2). As shown in the Fig. 8c sub-table, CWRs increase rapidly as CPA concentration decreases thereby also showing the need for faster warming techniques such as from metal forms.

## Discussion

This study demonstrates a new approach to increase the warming rates of vitrified biomaterials (solutions or tissues) using inductive heating of thin metal forms. The rates achievable are in excess of 1000 °C/min and hence far exceed the critical warming rates of numerous common CPAs. Indeed, the warming rates can be controlled by both choice of metal (type, amount and distribution), and field (frequency and magnetic field intensity) of the RF system. This in turn allows for the eventual rescue of samples that are sub-optimally loaded with CPAs and/or the use of lower concentration CPAs such as DP6. Furthermore, it suggests an opportunity to design the mechanical stress history of the sample to avoid cracking failures as recently explored within computational studies of inductively warmed samples.^5^,^29^

The ability to heat with metal forms is reliant on induced currents in the metal that do not distribute uniformly based on an effect called “skin depth.” In practice, over 98% of the current flow, and thus the bulk of the heating, will occur within a layer four times the skin depth from the surface. Thus, to increase the efficacy of heating, the thickness of the metal forms should be designed as ≥ 4 times the skin depth at the frequency of tuning. Otherwise the induced eddy currents will cancel each other that results in reduced heating as described further in the Supplemental Material. A summary of skin depth of different metals used in this study is given in Table 1. In brief, metal type, size and shape (i.e., thickness according to skin depth) and frequency of RF field operation are all important in ultrarapid warming.

Although convection is routinely used on small volumes (< 3 mL),^1^,^2^ the ability to use convection in larger systems will eventually fail due to cracking as the thermal stress continues to rise with size to be above the yield stress. However, properly designed ultra-rapid warming should be able to achieve uniform and fast warming within a few millimeters from the metal form regardless of the size of the system. This approach is likely to find utility in large-cell suspension or tissues that have lower concentrations of CPA after diffusive loading either due to the lower concentration of the CPA cocktail (i.e., DP6) or due to the increased thickness of the tissue (i.e., aorta).

As an example diffusive loading of VS55 into an artery only 0.8-mm thick achieves only 6 M at the centerline after 3 h.^18^ This physical study was much longer than the conventional loading protocols, which are closer to 75 min to avoid toxicity.^1^,^2^ Therefore, in practice, the center of boundary-loaded tissues will likely require faster warming rates than currently reported for the CPA alone. This will be particularly important in 2-mm thick aortic tissue (Fig. 7) and other tissues which are routinely 1.5–3.5-mm thick and are known to poorly load with CPA.^28^

Thus, a key advantage of ultrarapid warming is both the low cost of metal forms and the high achievable rates of over 1000 °C /min. For example, SAR from iron oxide nanoparticles for nanowarming are usually 160 W/g Fe at a cost of dollars/mg nanoparticles, whereas the SAR from metal forms ranges from 80 to 1000 W/g at estimated cost for aluminum foil of only cents/g metal. The metal forms can also be designed for easy removal in regular planar (e.g., heart valve, cartilage, suspension) and annular (e.g., artery) geometries.

It is important to mention that the biocompatibility of the metal used in this technique will be important when deployed in large-scale cell suspensions or in proximity to tissue surfaces. Various coating approaches may be needed to address this. Furthermore, although ultrarapid warming technology can be a good alternative for warming of tissues with surfaces and luminal structures, the need for nanowarming for vascularized bulk biomaterial at present cannot be addressed by distributed heating other than by deployment of nanoparticles within the vascular system.

## Electronic supplementary material

Below is the link to the electronic supplementary material.

Supplementary material 1 (DOCX 264 kb)
