# Supplementary material for: Ultrarapid Inductive Rewarming of Vitrified Biomaterials with Thin Metal Forms
Source: Ann Biomed Eng. 2018 Jun 19;46(11):1857–69. doi: 10.1007/s10439-018-2063-1 (PMC6208886; doi:10.1007/s10439-018-2063-1)
Supplement: Supplementary file 1 — Supplementary material 1 (DOCX 264 kb) [file 10439_2018_2063_MOESM1_ESM.docx]

**Supplemental Materials:**

CPA solutions: Euro-Collins, DP6, VS55

Euro-Collins (EC) is the carrier solution for VS55 and DP6. A 5× stock solution of EC was made by adding 174.76 g Dextrose, 10.2 g KH_2_ PO_4_, 36.5 g K_2_ HPO_4_, 5.6 g KCl, 4.2 g NaHCO_3_ in sufficient water to make up to 1 L as previously described [2], [18]. This 5x solution was then diluted 1:5 with water to make 1x solution where the pH and osmolality (mOsm) were adjusted to ~7.4 and 365 ± 5 respectively before storage at 4 °C until use.

DP6 is a cocktail comprised of 234.4 g/l DMSO (3M), 228.3 g/l propylene glycol (3M), and 2.4 g/l HEPES in 1x Euro-Collins solution [10], [20]. VS55 is made up of 242.14 g/l DMSO (3.1M), 168.38 g/l propylene glycol (2.2M), 139.56 g/l formamide (3.1M), and 2.4 g/l HEPES in the 1 x Euro-Collins solution. Fresh cryoprotectant solutions were prepared for each day of experiments.

Skin depth:

An alternating electric current will not distribute evenly within a conductor, leading to the highest current density near the surface which comprises a “skin-depth” effect, which is described in **Figure S1**. When a metal conductor is exposed to electromagnetic waves propagating in Z direction (normal to surface), the current density (J) can be based on Maxwell’s equation and described as:

$J={(\omega\mu\sigma)}^{1/2}e^{-z/\delta}\cos\left( \omega t+\frac{z}{\delta}-\varphi\right)H_{0}$ (S1)

where $\varphi$ is the phase angle between the electric and magnetic fields, $\omega$ is the angular frequency, $\sigma$ is the electrical conductivity, $\mu$ is the magnetic permeability, and $H_{0}$ represents the incident magnetic field. At a distance equal to one penetration depth below the surface ($z=\delta)$ the current density is falls to 1/e (~0.37) of its surface value. However, the power density will decrease to 1/e^2^ or 0.14 of its surface value. From this it can be concluded that almost 63 percent of the current and 86 percent of the power in the metal piece is concentrated within a surface layer of thickness $\delta$ which can be represented as:

$\delta=\sqrt{\frac{2\rho}{\omega\mu}}\sqrt{\rho\omega\varepsilon+\sqrt{1+{(\rho\omega\varepsilon)}^{2}}}$ , (S2)

where $\varepsilon=\varepsilon_{r}\varepsilon_{0}$, $\varepsilon_{r}$ is relative permittivity of the material, $\varepsilon_{0}$ is the permittivity of free space, $\mu=\mu_{r}\mu_{0}$, $\mu_{0}$ is the permeability of free space, and $\mu_{r}$ is the relative magnetic permeability of the conductor. At frequencies, where f <<1/$\rho\varepsilon$ the formula could be simplified from Maxwell’s equation to:

$\delta=\sqrt{\frac{\rho}{\pi f\mu_{0}\mu_{r}}}\approx503\sqrt{\frac{\rho}{f\mu_{r}}}$ (S3)

*Optimal frequency and metal piece selection:*

The frequency of the inductive current determines (resonance frequency of the coil) the depth that the induced eddy currents penetrate into the metal. For optimal heating, one important question is the optimal frequency at which a specific metal size of a specific type should be heated. Physically, the equivalent resistance (and thus the efficacy) of the metal is a function of the metal diameter d over the skin depth $\delta$ increasing rapidly up to ~ d/$\delta$=4. This means that if the coil frequency is fixed, then the metal diameter should be determined by this formula based on the material of metal forms. Or if the metal diameter is fixed by the application, then d/$\delta=4$ ratio will determine the working frequency. This frequency is called critical frequency. If the coil is operating below its critical frequency (f<f_cri_), the heating efficacy will be reduced due to eddy current cancellation from either side of metal forms. Increasing the frequency beyond the critical value (f>f_cri_) has very little effect on the relative efficacy. So ~ d/$\delta$=4 ratio is the rule of thumb to design thermal-seed inductive heating at a specific frequency of specific type of material.

Physically, the equivalent resistance of the metal, which determines the heating in a given field, is a function of the metal dimension perpendicular to the field. Due to skin-depth consideration, this dimension should be chosen such that $\frac{r}{\delta}\geq4$ at a given applied RF frequency (**Figure S1**), where δ and r are skin depth and ½ thickness of the metal seed, respectively.

Theoretical SAR in a cylindrical metal

Power generation in a cylindrical metal is found by calculating the spatially averaged absorbed power density of *P_I_* induced by a 1-cm long ferromagnetic rod of radius $a$, conductivity $\sigma$, and permeability $\mu$ [30], [31]. Neglecting the heat loss at the cylinder ends *P_I_* units are10^-2^ W/cm^3^ of system heated, and can be written as:

$P_{I}=\frac{\bar{P}_{cyl}\left( L=1cm \right)}{1{cm}^{3}}=\frac{\pi a}{\sqrt{2}}{(\frac{\omega\mu}{\sigma})}^{1/2}H_{0}^{2},$ (S4)

where $\sigma$ and $\mu$ are the conductivity and permeability of metal as shown for a variety of metals used in this study in **Table 1**. Here, volumetric SAR (W/m^3^) is calculated for a solid rod with the diameter of .965 mm heated in an RF system working at a frequency of 360kHz and magnetic field intensity (H_o_) of 20kA/m. Both volumetric and mass-based SARs for the metals used are reported in **Table 2**.

Heat transfer modeling and experiments:

The following conditions are applied to equation 2 and solved in COMSOL:

$\frac{\partial T(r,z)}{\partial r}=0\mathrm{at} r=0$ (S5)

$\frac{\partial T(r,z)}{\partial z}=0\mathrm{at} z=l$*_2_* (S6)

$-k\frac{\partial T(r,z)}{\partial z}=h\left( T(r,z)-T_{\infty} \right)\mathrm{at}r=l$*_1_* (S7)

$-k\frac{\partial T(r,z)}{\partial r}=h\left( T(r,z)-T_{\infty} \right)$at *z = 0* (S8)

$T_{i}(r,z)=-140℃$ , (S9)

where h is the convective heat transfer coefficient assumed to be 8 W/m^2^. This is an appropriate assumption for natural convection at these temperature ranges with environmental room temperature set at T=25°C. In case of convective warming in a water bath, the convective heat transfer coefficient of water is set as 100 W/m^2^K. Other properties used in these simulations are listed in **Table S2A** [5, 14, 33].

Both numerical and experimental cooling and ultrarapid warming results for other metal forms in VS55 including nitinol mesh and aluminum foil is shown in **Figure S2.** The system was cooled down at ~10°C/min, which is higher than the critical cooling rate needed for VS55 (2.5°C/min) as shown in **Figures S2A, S2B.** Similar to the foam, the slower controlled cooling method led to minimal thermal gradient in the sample for both metal forms.

The ultrarapid warming for these two foams show that both metal forms are able to reach warming rates much higher than CWR of VS55 (50°C/min). Specifically, nitinol mesh reached a warming rate of 330°C/min, while aluminum foil reached a warming rate of 1000°/min. The RF coil was shut off at -20°C for both these samples to avoid overheating of sample. There is a significant gradient in the data that is a result of the metal forms only being distributed close to the wall in a cylindrical fashion, leading to the wall being warmed up faster than the center. Nevertheless, both metal forms were able to achieve cooling and warming far faster than the CCR and CWR of VS55.

Solid mechanics modeling

The creep or viscous strain rate is defined as below and solved in COMSOL:

$\dot{\varepsilon}_{creep}=\frac{\sigma}{3\eta}$ (S10)

where $\sigma$ is the local stress and $\eta$ is the viscosity of the material. The elastic strain rate is defined as

$\dot{\varepsilon}_{elastic}=\frac{1}{E}\frac{d\sigma}{dt}$ (S11)

where E is the Young’s modulus of the CPA. And finally, the thermal strain rate is defined as

$\dot{\varepsilon}_{thermal}=\beta\frac{dT}{dt}$ , (S12)

where $\beta$ is the volumetric thermal expansion coefficient of the CPA similar to the method used in [5].

CPA loading diffusion model:

*Analytical Solution*

The solution in the main text of equation (4) with initial conditions and boundary conditions as noted below can be solved to a closed-form expression as follows. First, the non-homogeneity of boundary conditions can be removed by linearly shifting the temperature scale, namely defining a new temperature:

$\theta(r,t)=C(r,t)-C_{0}$ (S13)

The PDE, initial and boundary conditions become:

$\frac{1}{D}\cdot\frac{\partial\theta}{\partial t}=\frac{1}{r}\cdot\frac{\partial}{\partial r}(r\cdot\frac{\partial\theta}{\partial r})$ (S14)

I.C.: $\theta(r,0)=-C_{0}$ (S15)

Where C_0_ is the concentration loaded on the boundary (i.e. the solution outside of the tissue).

B.C.:$\left\{\begin{matrix} \theta(a,t)=0 \\ \theta(b,t)=0 \end{matrix}\right.$ (S16)

Assume a separation of *θ*(*r, t*) into space-dependent and time-dependent functions of a single variable each, namely,

$\theta(r,t)=R(r)\cdot\Gamma(t)$ (S17)

Where R(r) and Γ(t) can be solved as ordinary differential equations by the method of separation of variables and boundary conditions. Finally, the full partial differential equation can be solved by combining R(r) and Γ(t) and using orthogonality and initial conditions to construct the exact solution as outlined in Hahn & Ozisik [13]:

$\theta(r,t)=\sum_{n=1}^{\infty} C_{n}[J_{0}(\lambda_{n}r)Y_{0}(\lambda_{n}a)-J_{0}(\lambda_{n}a)Y_{0}(\lambda_{n}r)]\cdot e^{-D\lambda_{n}^{2}t}$ (S18)

Where the constant C can be solved for below and F(r) is the initial condition.

$C_{n}=\frac{\int_{r=a}^{b} r\cdot F(r)\cdot[J_{0}(\lambda_{n}r)Y_{0}(\lambda_{n}a)-J_{0}(\lambda_{n}a)Y_{0}(\lambda_{n}r)]\cdot dr}{\int_{r=a}^{b} r[J_{0}(\lambda_{n}r)Y_{0}(\lambda_{n}a)-J_{0}(\lambda_{n}a)Y_{0}(\lambda_{n}r)]^{2}\cdot dr}$ (S19)

We then substitute equation (S18) in (S13), yielding the result of the concentration inside the tissue at any location at any time:

$C(r,t)=C_{0}+\sum_{n=1}^{\infty} C_{n}[J_{0}(\lambda_{n}r)Y_{0}(\lambda_{n}a)-J_{0}(\lambda_{n}a)Y_{0}(\lambda_{n}r)]\cdot e^{-D\lambda_{n}^{2}t}$ (S20)

**Supplemental Figures and Tables:**

**Figure S1. Skin-depth effect in metal foam, foil or mesh.** In an alternating magnetic field (B fields), electrical current (E fields) produce currents (eddy currents) which oppose the direction of passing current through magnetic coil. These currents, I(r), do not distribute evenly within a conductor and are largest near the surface of a conductor (**A**). At a distance called the “skin depth”, equal to one penetration depth below the surface, current I(r) is seen to fall to 1/e (~0.37) of its surface value, I_o_. The skin-depth effect reduces with frequency increase (**B**).


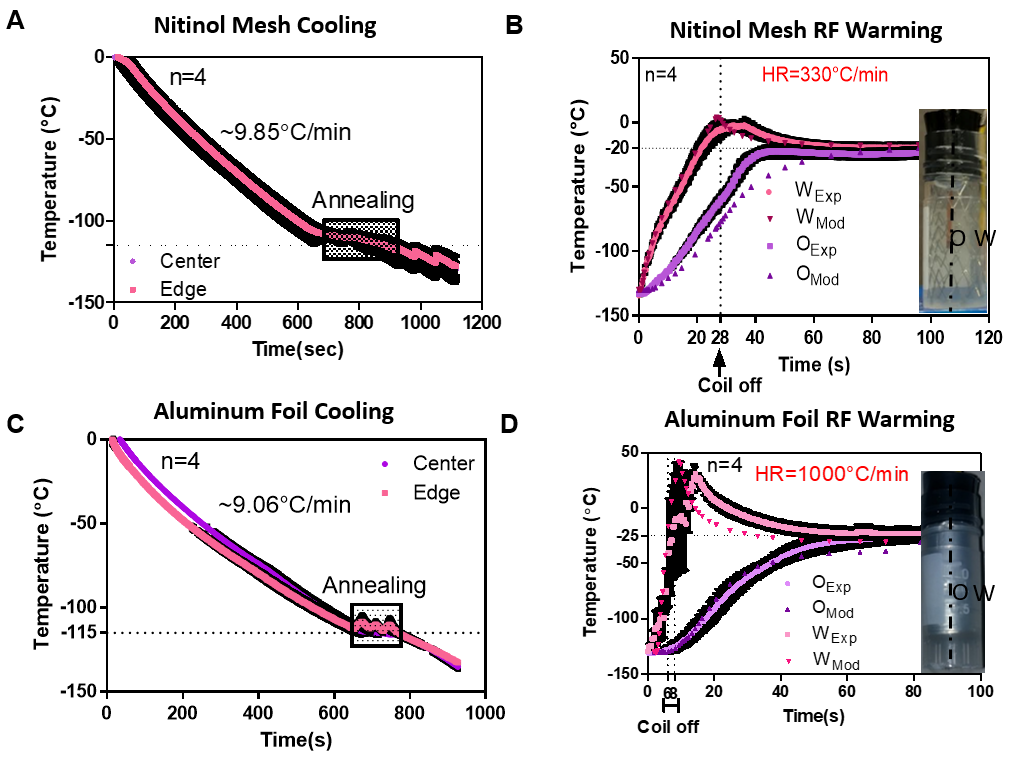


**Figure S2. Experimental and numerical measurement of cooling and ultrarapid warming rates of additional metal forms in VS55.** The cooling response of VS55 and nitinol mesh **(A)** or aluminum foil **(C)** implanted cryovials are shown. The cooling rates achieved in both cases exceed the CCR of VS55 (2.5°C/min). Experimental and numerical model ultrarapid warming responses for nitinol mesh **(B)** and aluminum foil **(D)** are also shown. Warming exceeds CWR of VS55 (50°C/min) in both cases.

**Table S1.** Metal RF Heating Parameters.

| Metal | $\mu$  $({\mu H}/m)$ | $\rho$  $\left( \muΩ.m \right)$ | Frequency | 1  kHz | 5  kHz | 30  kHz | 100  kHz | 360  kHz | 480  kHz |
| --- | --- | --- | --- | --- | --- | --- | --- | --- | --- |
| Copper | 1.25663 | 0.00168 | Skin depth (mm) | 2063 | 923 | 377 | 206 | 109 | 94 |
| Aluminum | 1.25665 | 0.00265 |  | 2591 | 1159 | 473 | 259 | 136 | 118 |
| Iron | 6300 | 0.00971 |  | 70 | 31 | 12.8 | 7 | 3.7 | 3.2 |

**Table S2A**. Thermal properties used in simulations.

| VS55 | $\left\{ \begin{aligned} 7.69T+2038 \\ 5.05T+2410 \\ 11.12T+2684 \end{aligned} \vert\begin{aligned} -119℃>T \\ -119℃\leq T<-45℃ \\ -45℃\leq T \end{aligned} \right\}$  [8] | $0.1065\times T+1062.8$  [8] | $0.3$  [8] |
| --- | --- | --- | --- |
| DP6 | $7.34T+1064 \left( T in K \right)$  [33] | $3.9\times{10}^{-7}T^{2}-1.9\times{10}^{-5}T-2.8\times{10}^{-2}\left( T in K \right)$  [33] | $2135T^{1.235}-1.893 T\leq273 K$  $0.2 T>273K$  [33] |
| Polypropylene | $3.936T+1121.1 \left( T in ^{\circ}C \right)$  [6] | $1075$  [6] | $-0.01274T+0.02 (T in ^{\circ}C)$  [6] |
| Material | Specific Heat  (J/kg K) | Density  (kg/m^3^) | Thermal Conductivity  (W/m K) |

**Table S2B**. Mechanical properties used in simulations.

| VS55 | $\left\{ \begin{aligned} 1.21\times{10}^{4} \\ \\ 4.2783\times{10}^{-23}e^{-0.6091T} \\ \\ 4.63\times{10}^{14} \end{aligned} \vert\begin{aligned} -100℃>T \\ \\ -140℃\leq T \\ <-100℃ \\ \\ T\geq-140℃ \end{aligned} \right\}$  [36] | $1.1\times{10}^{-4}$  [6] | $800\times{10}^{6}$  [6] | 0.25  [6] |
| --- | --- | --- | --- | --- |
| DP6 | $\left\{ \begin{aligned} 5.11\times{10}^{4} \\ \\ 5.6503\times{10}^{-23}e^{-0.6207T} \\ \\ 4.82\times{10}^{14} \end{aligned} \vert\begin{aligned} -100℃>T \\ \\ -137℃\leq T \\ <-100℃ \\ \\ T\geq-137℃ \end{aligned} \right\}$  [21] | $\left( 0.7798T+193.5 \right){10}^{-6}$  $\left( T in K \right)$  [33] | $1000\times{10}^{6}$  [33] | 0.2  [33] |
| Polypropylene | $N/A$ | $0.7\times{10}^{-4}$  [6] | $3.45\times{10}^{9}$  [6] | 0.35  [6] |
| Material | Viscosity  (Pa s) | Thermal Expansion Coefficient  (1/°C) | Young’s Modulus  (Pa) | Poisson’s Ratio |
